# Deep Reduced PEDOT Films Support Electrochemical Applications: Biomimetic Color Front

**DOI:** 10.3389/fbioe.2015.00015

**Published:** 2015-02-11

**Authors:** Jose G. Martinez, Beatriz Berrueco, Toribio F. Otero

**Affiliations:** ^1^Center for Electrochemistry and Intelligent Materials, Escuela Técnica Superior de Ingeniería Industrial, Universidad Politécnica de Cartagena, Cartagena, Spain

**Keywords:** conducting polymers, electrochromic front, redox reactions, ionic conductivity, reduced films

## Abstract

Most of the literature accepts, despite many controversial results, that during oxidation/reduction films of conducting polymers (CPs) move from electronic conductors to insulators. Thus, engineers and device’s designers are forced to use metallic supports to reoxidize the material for reversible device work. Electrochromic front experiments appear as main visual support of the claimed insulating nature of reduced CPs. Here, we present a different design of the biomimetic electrochromic front that corroborates the electronic and ionic conducting nature of deep reduced films. The direct contact PEDOT metal/electrolyte and film/electrolyte was prevented from electrolyte contact until 1 cm far from the metal contact with protecting Parafilm^®^. The deep reduced PEDOT film supports the flow of high currents promoting reaction induced electrochromic color changes beginning 1 cm far from the metal-polymer electrical contact and advancing, through the reduced film, toward the metal contact. Reverse color changes during oxidation/reduction always are initiated at the film/electrolyte contact advancing, under the protecting film, toward the film/metal contact. Both reduced and oxidized states of the film demonstrate electronic and ionic conductivities high enough to be used for electronic applications or, as self-supported electrodes, for electrochemical devices. The electrochemically stimulated conformational relaxation model explains those results.

## Introduction

Conducting polymers (CPs) submitted to electrochemical reactions have been proposed as a very simple material model (reactive macromolecules, ions and solvent) of the intracellular matrix from living cells (Otero and Martinez, 2013a). Driven by electrochemical reactions they originate biomimetic devices such as artificial muscles and actuators, electrochromic windows (UV-vis or IR), fast charge/discharge batteries, or supercapacitors mimicking electric organs or new artificial chemical synapses (Otero et al., 2012). Designing such biomimetic devices requires conductive (electronic and ionic) materials. Designers and development engineers approaching to those materials envisaging new applications realize that most of the literature asserts the insulating nature of films of conducting polymers in its reduced state claimed by the conducting/insulator transition model (Ofer et al., 1990; Aoki and Kawase, 1994; Zykwinska et al., 2003; Heinze et al., 2010). Different designs of the electrochromic front border show that the oxidation of deep reduced electrochromic films supported by a glass always starts at the polymer–metal interface used for the film connection with the electrical generator (Tezuka and Aoki, 1989; Tezuka et al., 1995, 1996). The final conclusion is that the reduced film is an insulator forcing the reaction beginning only through those polymer chains in direct contact with the metal. In those designs, the ensemble glass, polymer film, and metal are immersed inside the electrolyte. As final consequence reduced self-supported films of those materials are discarded as basic component of the above-mentioned devices and as electronic conductors for electrochemical purposes or devices.

Different experimental results contradict the insulating nature of deep reduced films of CPs. High spin (Petr and Dunsch, 1996; Zykwinska et al., 2003; Osterholm et al., 2008b) and charged states (Osterholm et al., 2008a) content in reduced films were detected by EPR or Raman spectroscopic studies. Full polymeric electrochromic devices, not including any metal contact inside the device, have been developed (Invernale et al., 2010). The conductivity of freestanding polypyrrole films reduced at high cathodic potentials for long times keeps high counterion content and electronic conductivities over 10^−3^ S cm^−1^ measured in inert atmosphere (Otero and Ariza, 2003; Otero and Martinez, 2014b). Deep reduced films support metal electrodeposition from aqueous solutions with flow of high current densities (Otero and Ariza, 2003). Freestanding films of CPs can be reduced by slow potential sweeps up to high cathodic potentials (more cathodic that −2V) in different electrolytes (solvents and salts) and then reoxidized during the subsequent anodic sweep getting stationary voltammetric responses (Otero et al., 2014). Artificial muscles constituted by freestanding films on isolating flexible tapes (Otero et al., 1993), or by interpenetrated polymer networks (Plesse et al., 2005) or by freestanding bilayer of two CPs (Kaneto et al., 1995) also support stationary voltammetric cycles from the reduced (supposed isolating) state to the oxidized states giving reverse bending movements.

The Electrochemically Stimulated Conformational Relaxation (ESCR) model states (Otero and Angulo, 1993; Otero et al., 1995, 1996, 1997; Grande and Otero, 1998; Otero and Padilla, 2004), and the Structural Chemical Kinetics (SCK) (Otero and Martinez, 2013b) corroborates that the film oxidation/reduction induces molecular (conformational) and macroscopic (swelling, shrinking, compactions, and relaxation) processes. After reaction induced structural closing the packed material traps over 20% of the counterions involved in the film redox processes, as proved by EDX analysis (Otero and Martinez, 2014b), which only can be expulsed very slowly through the packed film at high reduction overpotentials. Thus, after a deep reduction at high cathodic potentials any CP film keeps counterion and balancing polaron concentrations high enough to give electronic conductivities higher than 10^−3^ S cm^−1^.

The electrochromic front methodology was designed to support the isolating and porous nature of deep reduced CPs (Tezuka and Aoki, 1989; Tezuka et al., 1995, 1996). Films from oligomeric solutions were casted on an insulating support, as glass. A metal film (sputtered or by simple contact) allows the electrical contact at the film top. The full system (a fraction of the metal, the metal/polymer contact, and the polymer film) was immersed in the electrolyte. After an anodic potential step the oxidation induced color change starts at the metal/polymer interface and the electrochromic front advances toward the film bottom. Ulterior designs from Smela’s group, protecting now the film surface from the electrolyte contact letting the lateral borders free to contact the electrolyte, indicate that always the film reaction and the color change start at the electrode borders advancing toward the electrode center (Wang et al., 2004; Wang and Smela, 2009a,b).

In order to clarify this controversy, we will re-design here the electrochromic front experiment.

## Materials and Methods

**Figure 1:**
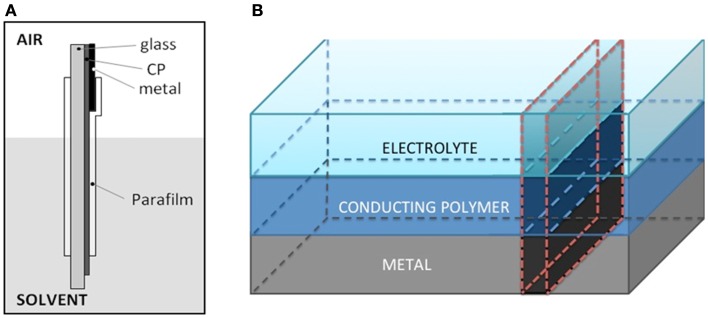
**(A)** Scheme of the new electrochromic front border design. The conducting polymer PEDOT–PSS film was casted on glass (2 cm^2^). A Pt foil having 1 cm^2^ of surface area allows the metal/CP electrical contact at the top. The Pt/polymer contact and most of the PEDOT film were surrounded with two Parafilm^®^ layers under strain to prevent the direct electrolyte contact. Around 1 mm of the CP remains uncoated at the bottom allowing there the direct CP/electrolyte contact. **(B)** The new design mimics a transversal cut of a conducting polymer (CP) film: metal/CP/electrolyte.

**Figure 2:**
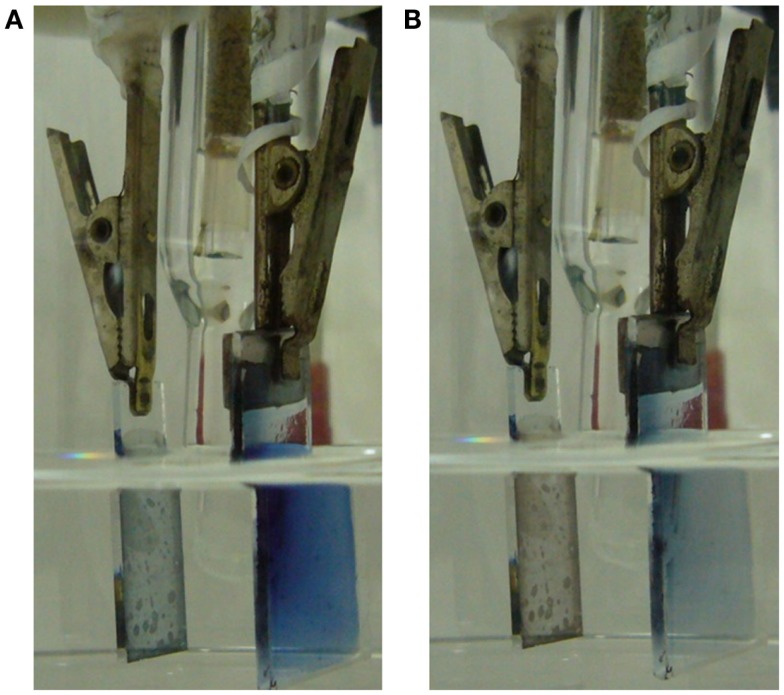
**Experimental cell: left side ITO counterelectrode, right side PEDOT–PSS film casted on glass showing a transversal Pt foil on the top allowing the clamp electrical contact**. A half of the Pt foil and most of the conducting polymer was coated with two layers of transparent Parafilm^®^. **(A)** Reduced PEDOT–PSS film. **(B)** Oxidized PEDOT–PSS film.

PEDOT/PSS films were obtained by evaporation from Aldrich aqueous solution on a glass plate of 2 cm^2^. In order to ensure the electrical contact, a platinum foil of 1 cm^2^ of area was put in contact with the polymeric film contacting 3 mm of the film top. The electrolyte–polymer contact was prevented in most of the film surface area by surrounding the ensemble under pressure and strain with two Parafilm^®^ layers. About 1 mm of the PEDOT/PSS film remains uncoated at the film bottom to allow the PEDOT contact with the electrolyte. At the top, 2 mm of the Pt foil keep uncoated to allow the electrical clamp contact (Figure 1A). The electrochemical cell was a transparent tank containing 0.2 M LiClO_4_ (Aldrich) aqueous solution. An ITO electrode (4 cm^2^) was used as counter electrode. The reference electrode was a Crison Ag/AgCl (3 M KCl). First the PEDOT/PSS was deep reduced at −1V for 5 min to guarantee a deep reduced initial state. Then it was submitted to consecutive square potential waves from −1.00 V for 10 s to 0.20 V for 10 s. Figure 2 shows two pictures of the cell with the oxidized electrode (transparent blue light color) and reduced electrode (blue dark color).

To ensure stationary responses, eight consecutive square potential waves were applied to the electrode. Color changes were recorded in parallel using a compact Sony video camera. Images were treated by Virtual Dub software and by ImageJ software to evaluate de the color gradient evolution between consecutive frames.

## Results and Discussion

The reduced PEDOT film is casted on an isolating and transparent glass (Figure 1A). At the top a Pt foil allows, by direct contact, the electronic contact and the current flow. The ensemble is surrounded, under strain and pressure, with a transparent Parafilm^®^ in order to protect most of the deep reduced film, the film–metal interface and most of the metal from the direct electrolyte contact. At the electrode top, 1 mm of uncoated Pt foil allows the electrical connection, through a metal clamp, with the generator. At the electrode bottom, 1 mm of unprotected PEDOT film allows the CP/electrolyte contact (Figure 1A). Thus the Parafilm^®^ protects from the electrolyte direct contact 10 mm, from the metal/PEDOT contact to the PEDOT/electrolyte contact, of the PEDOT film surface and lateral sides. This new design reproduces, at a larger scale, a theoretical transversal strip from the metal until the solution (Figure 1B) in the middle of a CP film coating a metal electrode. Here, the metal–polymer contact is far from the solution, furthermost than in any film coating a metal. Only a small fraction (1 mm) of the film remains uncovered at the film bottom (imitating the polymer/solution interface of the coated metal electrode). If the deep reduced film is an insulator requiring the metal/polymer contact inside the electrolyte to start the polymer re-oxidation both, film oxidation and color change will be inhibited.

**Figure 3:**
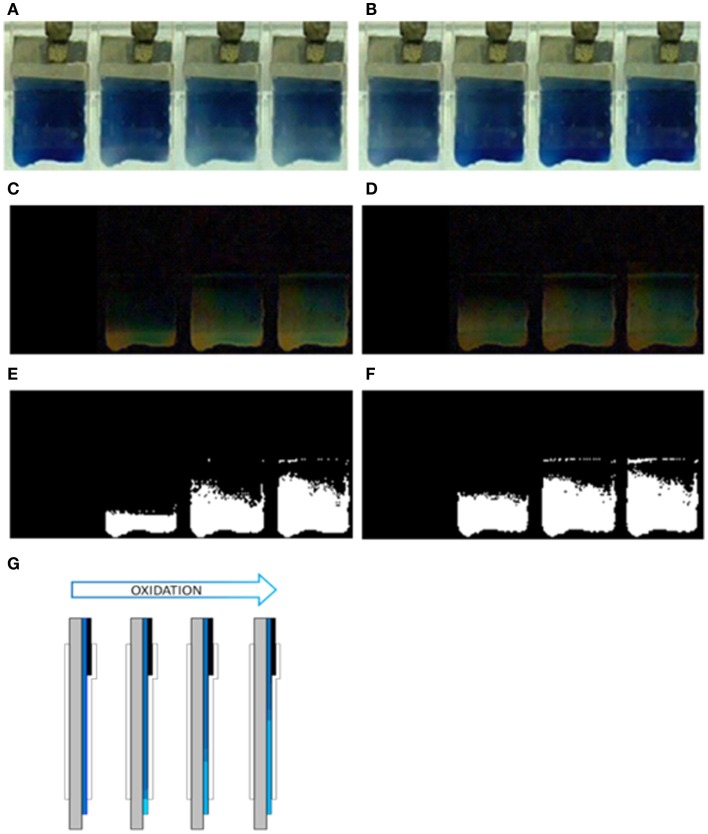
**Advance of the electrochromic front border through PEDOT–PSS films casted on glass in 0.2 M LiClO_4_ aqueous solution**. **(A)** Oxidation process at 0.20 V after 0, 2, 6, and 9 s. **(B)** Reduction process at −1.00 V after 0, 2, 6, and 10 s. **(C)** Image differences with that at time 0 s (using the image treatment program “ImageJ”) for the oxidation process **(A,D)** image differences for the reduction process **(B,E)** binarized images from **(C,F)** binarized images from **(D,G)** schematic representation of the electrochromic oxidation: the blue light color advances from the bottom by consuming the blue dark color.

Figure 2A shows the blue dark uniform color of the polymer film after reduction at −1.00 V for a long time. Figure 2B shows the blue light color of the polymer film inside the electrolyte after oxidation at 0.2 V for a long period of time. Starting from the deep reduced film, after a potential step from −1.00 to 0.20 V, the polymer oxidation, visualized by the color change from blue dark to blue light, is initiated (Figure 3A) at the electrode bottom narrow strip in direct contact with the electrolyte. The electrochromic front advances through the PEDOT film from the film bottom, underneath the transparent Parafilm^®^, toward the polymer/metal contact, Figure 3A from the left picture to the right picture.

Thus, the oxidation induced color change is initiated at the narrow PEDOT strip in direct contact with the electrolyte, at the electrode bottom, 1 cm far from the film/metal interface at the electrode top. The electrons extracted from the PEDOT strip chains during its oxidation must flow to the potentiostat through the 1 cm deep reduced film present between this strip and the metal contact. This result unambiguously indicates that the electronic conductivity of 1 cm of the dark blue and deep reduced film at −1V for long time supports the current flow required to initiate the PEDOT oxidation at the other film end: the reaction starts at the place where counterions, required for the reaction charge balance, are available from the solution. This oxidation drives the film color change from blue dark (reduced) to blue light (oxidized) and the oxidation progress drives the advance of the blue dark oxidized front by consumption of the reduced blue light film toward the polymer/metal contact. The color advance toward the metal contact underneath the Parafilm^®^ indicates that the ionic conductivity through the oxidized film from the solution toward the oxidized/reduced front is also high enough to allow the reaction progress.

By stepping the potential back to −1.00 V the color changes back to dark blue (reduced) and the new generated reaction front also advances from the film bottom to the metal contact (Figure 3B, from the left picture to the right picture). The ionic conductivity through the reduced film underneath the Parafilm^®^ toward the solution is also high enough to allow the advance of the reduced front.

The evolution of the front border is improved by color subtraction. Figures 3C,D were obtained by difference between the first image (showing the film at the beginning of the potential step), and each the subsequent ones. Figures 3E,F were obtained by binarization (transparent/black related to a threshold color) of Figures 3C,D.

The same results above described for the movement of the oxidized or reduced front is reproduced every time when the electrode is submitted to consecutive square potential steps: both, oxidation and reduction processes start, every time, at the polymer/electrolyte interface advancing underneath the Parafilm^®^ toward the polymer/metal interface. This stationary reproducibility also sustains that the conductivity of the deep reduced film is always high enough to allow the initiation of the polymer oxidation 10 mm far from the metal/polymer contact. If, as proposed by the conducting/insulator model the reduced polymer was an insulator, its re-oxidation at 10 mm from the polymer/metal contact should become prohibited.

Those results unambiguously corroborate that both states (oxidized and reduced) of the PEDOT film present high enough electronic and ionic conductivities to support film electrochemical reactions taking place far away from the metal contact. That means that engineers and designers can use self-supported films of CPs as electrodes for any electronic or electrochemical application (actuators and artificial muscles; batteries and supercapacitors; smart windows, glasses, or mirrors; smart membranes with tuned transversal ionic conductivity; and chemical storage for drug delivery or artificial chemical synapse, ionic sensors, biosensors and proprioceptive sensors, and so on) (Otero et al., 2012;Otero and Martinez, 2013a, 2014b).

The origin of the conducting/insulator transition model during the electrochemical reduction of CPs apparently comes from the theoretical calculations of the insulating nature of an isolated, ideal, and neutral (without any charge) chain of any CP. The relatively high electronic and ionic conductivity of deep reduced films here deduced or the contradictory results presented at the introduction: high spin states and charged states (EPR and Raman results), high concentration of counterions in deep reduced films (XPS), film reduction reaction going on up to very high cathodic potentials or different devices, as artificial muscles, only constituted by polymers giving stationary voltammetric responses (reduction and re-oxidation) at very low potential sweeps (to get a deep reduced state) up to −3.5 V, does not contradict the calculated insulating nature of neutral individual chains. According with the ESCR model (Otero et al., 1995, 1996, 1997), CPs relax, swell, shrink, and compact under oxidation/reduction control. Those structural changes are corroborated by determination of dimensional changes during oxidation/reduction (Otero and Martinez, 2014a), by its application to develop artificial muscles (Otero et al., 1992) or smart membranes, which transversal ionic flow can be tuned by the oxidation state (swollen or shrunk) of the film and by the SCK model (Otero and Martinez, 2013b). In films of CPs the oxidized material (polymer, balancing counterions, and solvent) presents a swollen structure. During reduction the materials, exchanging anions or cations, trap up to 30% of the counterions (and the balancing positive charges on the chains) inside the film (Otero et al., 2014). The reduction reaction rate becomes slower (Otero and Martinez, 2013b) going on up to very cathodic potential limits. Getting a full-reduced film (without any counterion inside) becomes a very difficult (for usual experimental times) task for films thicker than 0.5 μm. So, any reduced material keeps counterions and balancing polarons presenting a high electronic conductivity, as underlined in the literature. Getting lower electronic conductivities than 10^−4^ S cm^−1^ requires very long reduction times (days or weeks), at high cathodic overpotentials (more cathodic that −1V) and quite thin (<0.1 μm) films. According with the Ohm’s law, higher conductivities than 10^−4^ S cm^−1^ can support several milliampere per square centimeter of current flow and fast electrochemical reactions, as those observed in Figures 2 and 3.

**Figure 4:**
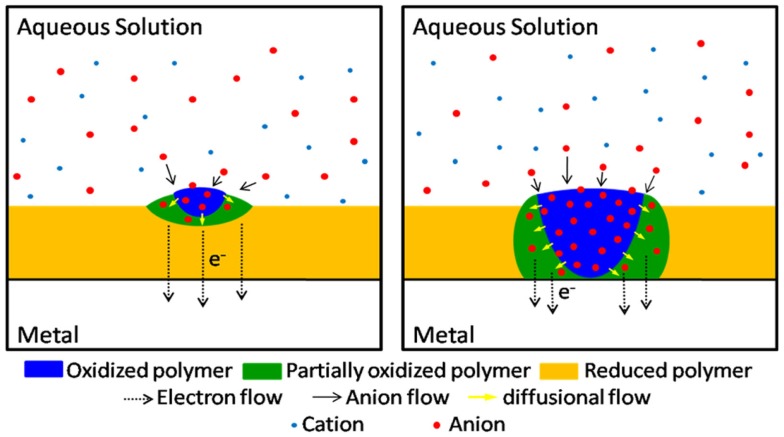
**Scheme of the oxidation according to the ESCR model: the oxidation of conformational packed and reduced film of any conducting polymer starts by nucleation–relaxation at the polymer/solution interface**.

The relatively high electronic conductivity of deep reduced films, and results from Figure 3, also give some light on other controversial point. Where the oxidation of a deep reduced film of a CP coating a metal starts? The chronoamperometric responses during re-oxidation present a large maximum (Otero and Boyano, 2003; Otero and Martinez, 2013b) indicating that the re-oxidation begins by nucleation of the oxidized material. Supporters of the conducting/insulator transition model advocate a nucleation starting at the polymer/metal interface of the porous polymer film. Thus extracted electrons flow to the metal from those insulating polymer chains touching it: by oxidation those chains become conducting, originating a reaction front that advances from the polymer/metal interface toward the polymer/electrolyte interface. Results presented in this paper unambiguously demonstrate that the oxidation of deep reduced films in contact with a metal begins at the polymer/electrolyte interface. We can conclude that the oxidation of reduced and compacted films is initiated by nucleation–relaxation (Figure 4) at the polymer/solution interface, as proposed by the ESCR model (Grande and Otero, 1998; Otero and Boyano, 2003; Otero and Padilla, 2004), advances toward the polymer/metal interface originating expanding cylindrical columns of oxidized polymer. From those expanding columns a good theoretical modelization of the electrochemical responses is attained: the ESCR model.

## Conclusion

Deep reduced films of PEDOT–PSS protected from the direct electrolyte contact present an electronic conductivity high enough to support the initiation of electrochromic changes, driven by oxidation/reduction reactions, at the polymer/electrolyte contact located 1 cm apart from the metal/film electrical contact. The electrochromic front advances, consuming the deep reduced film, from the film bottom to the polymer/metal contact at the electrode top. Those results support one of the hypothesis from the ESCR model: the oxidation of deep reduced films of CPs start at the polymer/electrolyte interface; in opposition to the conducting/insulator transition model stating that this oxidation begins at the polymer/metal interface of the porous (ionic conductor) and electronic insulator film, advancing from there toward the polymer/electrolyte interface.

An important technological consequence merges from the relatively high electronic and ionic conductivity of deep reduced films: they can be used as self-supported electrodes or as electronic conductors by engineers and designers to develop electronic or biomimetic electrochemical devices as artificial muscles and actuators; smart membranes tuning the transversal ionic flow by the membrane oxidation-swollen or reduced-packed state; smart drug (pharmaceutical, fertilizer, and neurotransmitter) deliverer; artificial chemical synapse; batteries and supercapacitors; smart windows, mirrors and glasses; sensors biosensors and proprioceptive sensors and devices; and so on.

## Author Contributions

The three authors participated in the conception of the experiments. Experimental work was performed by BB with help of JM and TO. All authors have participated in writing and have approved the final version of the draft. All authors agree to be accountable for all aspects of the work in ensuring that questions related to the accuracy or integrity of any part of the work are appropriately investigated and resolved.

## Conflict of Interest Statement

The authors declare that the research was conducted in the absence of any commercial or financial relationships that could be construed as a potential conflict of interest.
